# A Resonant Pressure Microsensor Capable of Self-Temperature Compensation

**DOI:** 10.3390/s150510048

**Published:** 2015-04-29

**Authors:** Yinan Li, Junbo Wang, Zhenyu Luo, Deyong Chen, Jian Chen

**Affiliations:** 1 State Key Laboratory of Transducer Technology, Institute of Electronics, Chinese Academy of Sciences, Beijing 100190, China; E-Mails: yzngb@163.com (Y.L.); oreilzy@gmail.com (Z.L.); dychen@mail.ie.ac.cn (D.C.); chenjian@mail.ie.ac.cn (J.C.); 2 University of Chinese Academy of Sciences, Beijing 100049, China

**Keywords:** pressure sensor, resonance, temperature self-compensation, MEMS

## Abstract

Resonant pressure microsensors are widely used in the fields of aerospace exploration and atmospheric pressure monitoring due to their advantages of quasi-digital output and long-term stability, which, however, requires the use of additional temperature sensors for temperature compensation. This paper presents a resonant pressure microsensor capable of self-temperature compensation without the need for additional temperature sensors. Two doubly-clamped “H” type resonant beams were arranged on the pressure diaphragm, which functions as a differential output in response to pressure changes. Based on calibration of a group of intrinsic resonant frequencies at different pressure and temperature values, the functions with inputs of two resonant frequencies and outputs of temperature and pressure under measurement were obtained and thus the disturbance of temperature variations on resonant frequency shifts was properly addressed. Before compensation, the maximal errors of the measured pressure values were over 1.5% while after compensation, the errors were less than 0.01% of the full pressure scale (temperature range of −40 °C to 70 °C and pressure range of 50 kPa to 110 kPa).

## 1. Introduction

Pressure microsensors are widely used in the fields of aerospace exploration and atmospheric pressure monitoring due to their advantages of small size, high resolution and low cost [1]. Based on their detection mechanism, these microsensors can be classified into capacitive pressure microsensors [2,3], piezoresistive pressure microsensors [4,5], piezoelectric pressure microsensors [6,7] and resonant pressure microsensors [8]. Compared to other types of pressure sensors, resonant pressure microsensors offer the advantages of “quasi-digital” output, which allows direct coupling to digital electronics without analog to digital converters, leading to high resolution and reliability. Additionally, resonant pressure micro-sensors featured long-term stability since the resonant frequencies are mainly determined by the intrinsic material properties and geometrical parameters [9,10].

Temperature disturbance is a key concern in the field of pressure microsensors [11]. More specifically, in the field of resonant pressure microsensors, temperature variations can cause stress changes in resonators, which lead to frequency drift in response to temperature variations. Thus, temperature is a critical factor in resonant pressure microsensors and various approaches have been proposed for the purpose of temperature compensation [12].

Methods enabling temperature compensation can be classified into two types, relying on hardware compensations and software compensations, respectively. Hardware compensations are usually realized by including additional temperature-sensitive components within the sensor module or adding temperature compensating elements in the detection circuit. Hardware compensations are simple to use, but however they suffer from the problem of low flexibility and thus low compensation accuracy [13]. To improve the compensation accuracy, software compensations have been proposed where microprocessors were used to process raw data obtained from additional temperature sensors based on appropriate compensation algorithms to address the issue the temperature drift [14,15,16]. However, these conventional compensation approaches rely on external temperature sensors for monitoring the surrounding temperature. Due to the temperature distribution uncertainty and the thermal conduction delay, these methods suffer from limited accuracy [17].

The concept of self-temperature compensation is based on the temperature characteristics of the sensors themselves, where additional temperature sensing elements are not needed to perform temperature compensations [18]. Wang *et al.* proposed a self-temperature compensation method which utilized the different temperature characteristics between the fundamental frequency and the third harmonic frequency of the same resonator [19]. Although this method uses a single resonator to achieve temperature self-compensation, the resonator cannot function in a closed-loop manner, which results in low accuracy and long response time.

Our previous work [20] proposed a differential output pressure microsensor with double “H” type resonators. Based on this double-resonator structure, a self-temperature compensation approach was put forward in this study, which reported an accuracy of ±0.01% over the full pressure and temperature scale (temperature range of −40 °C to 70 °C and pressure range of 50 kPa to 110 kPa). In this method, the compensation can be realized only after acquiring the frequencies of two resonators without the need of additional temperature sensors.

## 2. Device Structure

**Figure 1:**
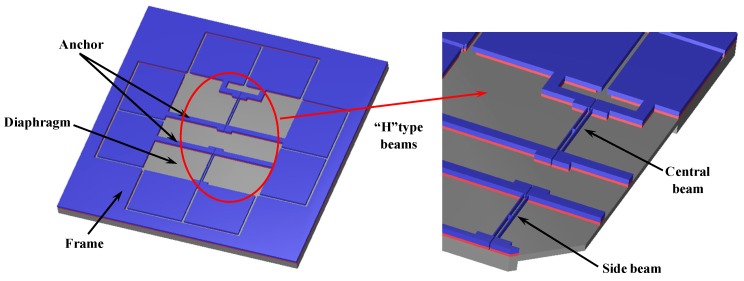
The schematic diagram of the differential pressure sensor includes a square pressure-sensitive diaphragm and two “H” type doubly-clamped resonant beams. The pressure under measurement causes a deflection of the diaphragm, which is translated to an axial tensile stress in the central beam and an axial compressive stress in the side beam, leading to resonant frequency shifts towards opposite directions as a differential output.

The schematic diagram of the differential resonant pressure microsensor is shown in Figure 1. The resonators consist of two “H” type doubly-clamped beams immobilized on a square pressure-sensitive diaphragm. These two resonators, which are actuated and detected electromagnetically, function in a lateral mode. The magnetic field is perpendicular to the diaphragm. When an alternating current is applied to a half beam (actuation beam) of the “H” type beam, the Ampere force will cause it to vibrate, while the vibration of the other half beam (detection beam) of the “H” type beam will cause an electromotive force, which allows us to measure the vibration frequency. The two “H” type beams, namely “central beam” (located in the center of the diaphragm) and “side beam” (located near the border of the diaphragm), respectively, have almost identical dimensions and thus comparable resonant frequencies at zero pressure loads [21,22].

In this device, the pressure under measurement causes a deflection of the diaphragm. According to elasticity theory, this deflection will produce tensile stress near the middle of the diaphragm and compressive stress near the edge of the diaphragm. The stress of the diaphragm is passed to the beams through the anchor, which results in a frequency increase of the central beam and a decrease in the side beam, leading to a frequency differential output.

Since the two beams have almost identical dimensions, the resonant frequencies of the beams drift in the same direction in response to temperature changes. Due to the differential design, this temperature-based resonant frequency drift can be partially suppressed. However, due to manufacturing uncertainties and position variations, the temperature drift of these two beams cannot be identical. Thus, a temperature compensation algorithm is required to further address the effect of temperature variations on the resonant frequency drift.

## 3. The Self-Temperature Compensation Algorithm

Based on the elasticity theory, the natural frequency of the resonant beam in the first-order vibration mode is [23]:

(1) $$f_{0}=f_{1}\left(0\right)=\frac{4.73^{2}}{2\pi l^{2}}\sqrt{\frac{EI}{A\rho }}=1.028\sqrt{\frac{E}{\rho }}\cdot \frac{b}{l^{2}}$$

where *E* is Young’s modulus of the material, *I* is moment of inertia, *A* is the cross-sectional area of the beam, ρ is the material density, *b* is the width of the beam, and *l* is the length of the beam. Among these parameters, *l* is related to the stress state of the beam, dominated by pressure (*P*) under measurement, while *E*, ρ, *b*, *l* are all related to temperature (*T*). Thus the resonant frequencies can be expressed as a binary function of pressure and temperature, which is:

(2) $$\left\{ \begin{array}{l} f_{1}=F_{1}(p,T)\\ f_{2}=F_{2}(p,T) \end{array}\right.$$

where *f*_1_ is the resonant frequency of the central beam, *f*_2_ is the resonant frequency of the side beam, *p* is the pressure load, and *T* is the temperature. According to Equation (2), pressure and temperature can be expressed as a binary function of *f*_1_ and *f*_2_ based on mathematical translation as follows:

(3) $$\left\{ \begin{array}{l} p=G_{1}(f_{1},f_{2})\\ T=G_{2}(f_{1},f_{2}) \end{array}\right.$$

where function *G*_1_ is the self-temperature compensation function.

According to Equation (3), the pressure *p* is determined by the two resonant frequencies, *f*_1_ and *f*_2_, which form a 3D surface at the coordinates (*f*_1_, *f*_2_). This function can be expressed by a polynomial surface fitting, which is:

(4) $$p=a_{0}+a_{1}f_{1}+a_{2}f_{2}+a_{3}f_{1}^{2}+a_{4}f_{1}f_{2}+a_{5}f_{2}^{2}+a_{6}f_{1}^{3}+a_{7}f_{1}^{2}f_{2}+a_{8}f_{1}f_{2}^{2}+a_{9}f_{2}^{3}+\varepsilon$$

where *a*_0_,…, *a*_9_ are coefficients, ε is higher-order infinitesimal.

In order to determine the coefficients in Equation (4), a calibration process for the sensor is required. *m* points were selected for temperature calibration in the full temperature range of −40 °C~70 °C, and *n* points were selected for pressure calibration in the full pressure scale of 50 kPa~110 kPa (atmospheric pressure in this work). In total, *m* × *n* points were used.

After calibration, *a*_0_,…, *a*_9_ can be calculated with calibration data based on the least square method. The mean square error *R* can be expressed as:

(5) $$R=\frac{1}{m\times n}\sum_{k=1}^{m\times n}{\left[p_{k}-p(f_{1k},f_{2k})\right]}^{2}$$

where *p_k_*, *f*_1k_, *f*_2k_ are calibration data, *k* = 1, 2, 3,…, *m* × *n*. In order to minimize *R*, partial derivatives of *R* with respect to *a*_0_,…, *a*_9_ should be equal to zero, which is:

(6) $$\frac{\partial R}{\partial a_{i}}=0,i=0,1,...,9$$

Thus, the coefficients, *a*_0_, …, *a*_9_, can be solved with Equation (6). By substituting these coefficients into Equation (4), the self-temperature compensation function can be obtained.

**Figure 2:**
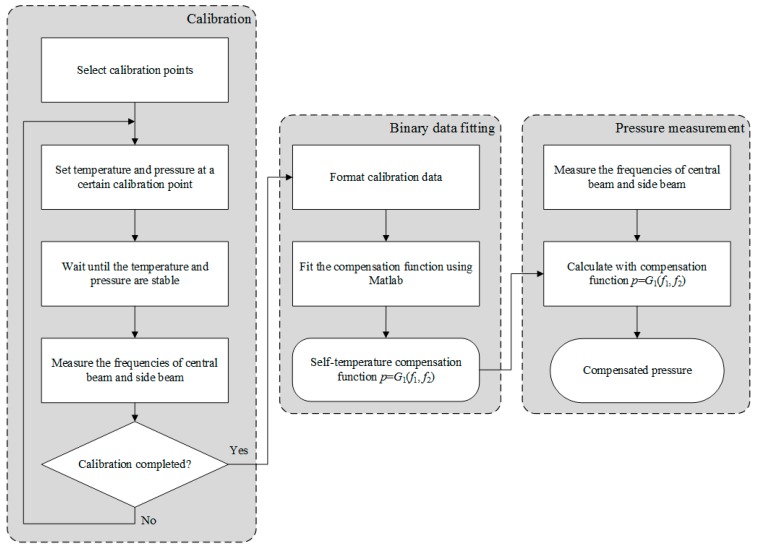
The self-temperature compensation flow chart includes three main steps: Calibration, binary data fitting, and pressure measurement. In the calibration step, the frequencies of the central beam and side beam were measured at each temperature and pressure point. These groups of data were fitted in binary data fitting step to obtain the self-temperature compensation function. Then this function was programmed into the post processing unit of the sensor in pressure measurement step to calculate the pressure in real time.

The complete self-temperature compensation process is summarized in Figure 2. As the first step, certain calibration points in the full temperature and pressure scales were selected with two resonant frequencies quantified. Secondly, polynomial surface fitting with the calibration data was conducted to obtain the self-temperature compensation function. Third, this compensation function was programmed into the post processing unit of the sensor, and thus the pressure microsensor can function in the of self-temperature compensation mode.

**Figure 3:**
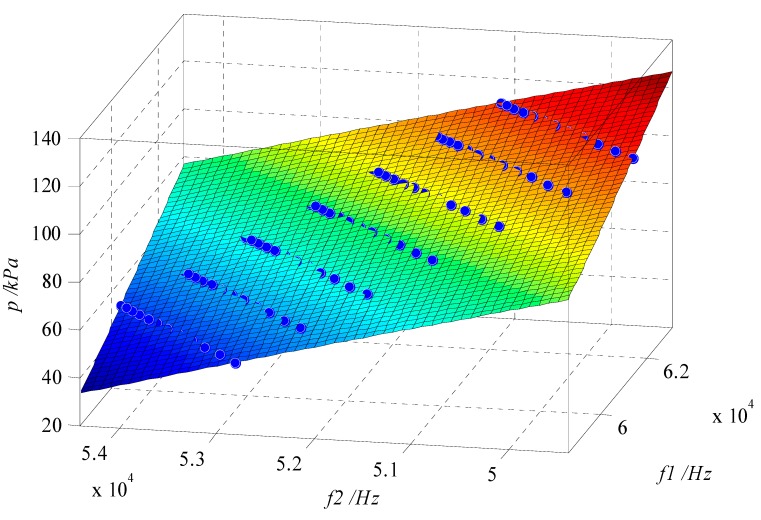
Surface plot of calculated pressure values obtained after cubic polynomial surface fitting as a function of two resonant frequencies *f*_1_ and *f*_2_. The blue dots indicate the calibration points.

In this study, 12 temperature calibration points were chosen in the range of −40 °C~70 °C (one point per 10 °C, regular value in calibration of atmospheric pressure sensors), and seven pressure calibration points were chosen in the range of 50 kPa~110 kPa (one point per 10 kPa). The temperature was controlled by a Temperature & Humidity Chamber (SH-241, ESPEC Co., Osaka, Japan), and the pressure was controlled by a Pressure Controller/Calibrator (PPC4, Fluke Co., Phoenix, AZ, USA). In addition, the resonant frequency was measured by a Digit Multimeter (2100 6 1/2, Keithley Co., Cleveland, OH, USA). The 3D surface plot of pressure in the coordinates (*f*_1_, *f*_2_) is shown in Figure 3.

## 4. Calibration Point Selection Optimization

According to the theory of the least square method, the proper selection of calibration points has a significant influence on the fitting result. The calibration in the whole pressure and temperature range for the sensor is time-consuming work. The procedure can be too complicated if the number of the calibration points is too high while the compensation accuracy may decrease if the number of the calibration points is too low. Thus, it’s necessary to properly select the calibration points to find a balance between accuracy and complexity. In addition, compared to the pressure calibration, the temperature calibration has a much higher degree of complexity due to the thermal conduction delay. Thus, the optimization was focused on choosing the number of temperature calibration points.

**Figure 4:**
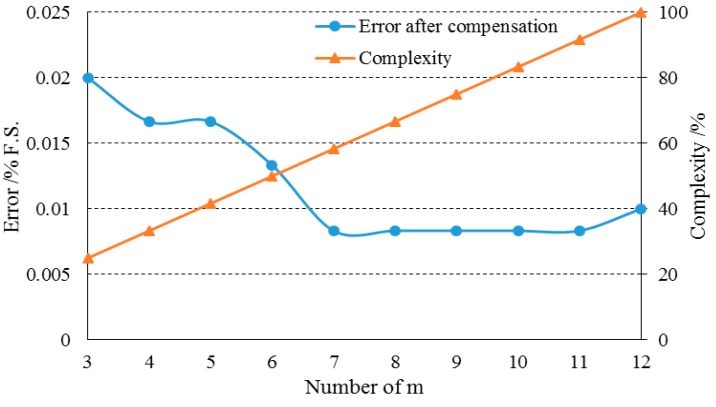
The selection of calibration points has an influence on the compensation error. M = 7 is the optimal calibration points of temperature, which maintains a high accuracy and reduces the complexity by 41% compared to the case of m = 12.

Figure 4 shows the result of the optimization, where *m* is the number of temperature calibration points in the range of −40 °C~70 °C, and error is the percentage absolute error between the calculated pressure value by compensation function and the calibration pressure value, and complexity is the percentage of the time required to complete the calibration process compared to that when *m* = 12. When *m* was decreased from 12 to 7, the maximal error basically remained unchanged. When *m* was further decreased to 6, an increase of the maximal error after compensation was observed. Based on this result, *m* = 7 is the optimized number for temperature calibration, which maintains a high accuracy and reduces the complexity by 41% compared to the original calibration procedure when m = 12.

## 5. Compensation Results and Analysis

**Figure 5:**
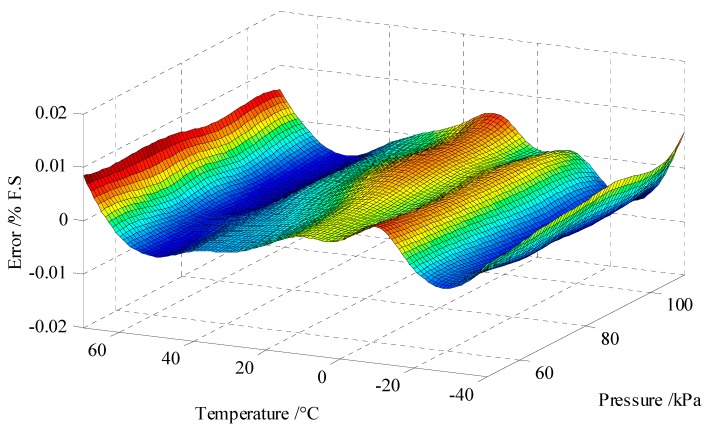
Surface plot of the pressure compensation error as a function of temperature and pressure, which is less than ±0.01% F.S. (pressure range of 50 kPa to 110 kPa and temperature range of −40 °C to 70 °C).

The difference (error) between the experimental results of *p_k_* and the compensated pressure value *p*(*f*_1*k*_, *f*_2*k*_) is represented by Δ*_k_*. The 3D surface plot of this error is shown in Figure 5. The compensation error was less than ±0.01% of full pressure scale (50 kPa~110 kPa) in the full temperature range (−40 °C~70 °C).

**Figure 6:**
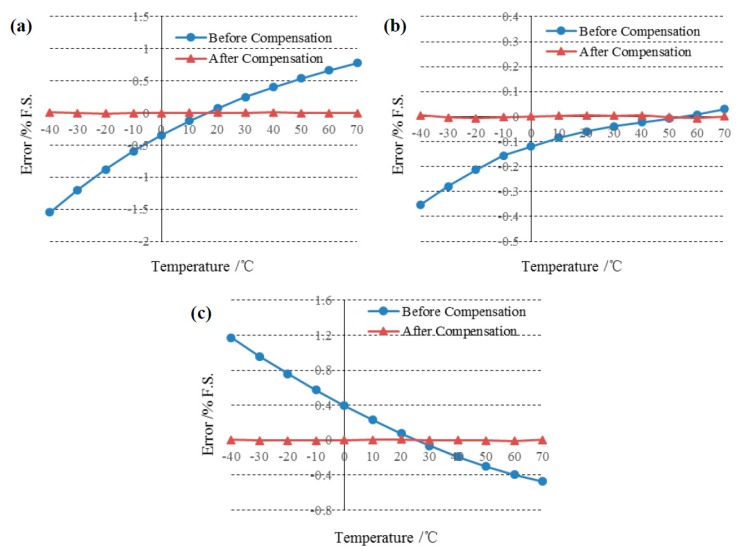
Comparison of the errors before and after compensation at the pressure of (**a**) 50 kPa; (**b**) 80 kPa and (**c**) 110 kPa. Before compensation, the max error was higher than 1.5% while after compensation, the errors were lower than 0.01%.

Comparisons of the errors of full pressure scale before and after temperature compensation are shown in Figure 6 where the temperature was changed from −40 °C to 70 °C at 50 kPa (Figure 6a), 80 kPa (Figure 6b) and 110 kPa (Figure 6c), respectively. Before compensation, the maximal error was higher than 1.5% when the sensor worked in the differential mode. After applying this self-temperature compensation algorithm, the errors were significantly decreased to less than 0.01%.

In addition, the sensors were tested at additional pressure and temperature values besides the calibration points, to further validate the functionality of the self-temperature compensation method. −15 °C, 10 °C, 25 °C and 50 °C were chosen as points for temperature testing and 50 kPa, 65 kPa, 80 kPa, 95 kPa, and 110 kPa were picked as points for pressure testing. The test results are shown in Table 1 where the quantified maximal error was −3.7 Pa, which was lower than ±0.01% F.S.

**Table 1:** The quantified errors at different pressure and temperature points. The maximal error measured was −3.7 Pa, which was lower than ±0.007% F.S.

Error/Pa		Testing Pressure/kPa				
		50	65	80	95	110
Testing temperature /°C	−15	1.7	1	−0.7	−0.8	−3.7
	10	0	−0.5	−0.2	−1.2	−1.9
	25	0.9	1.1	1.1	−0.2	−0.4
	50	1.3	1.2	0.8	−0.4	−0.7

**Figure 7:**
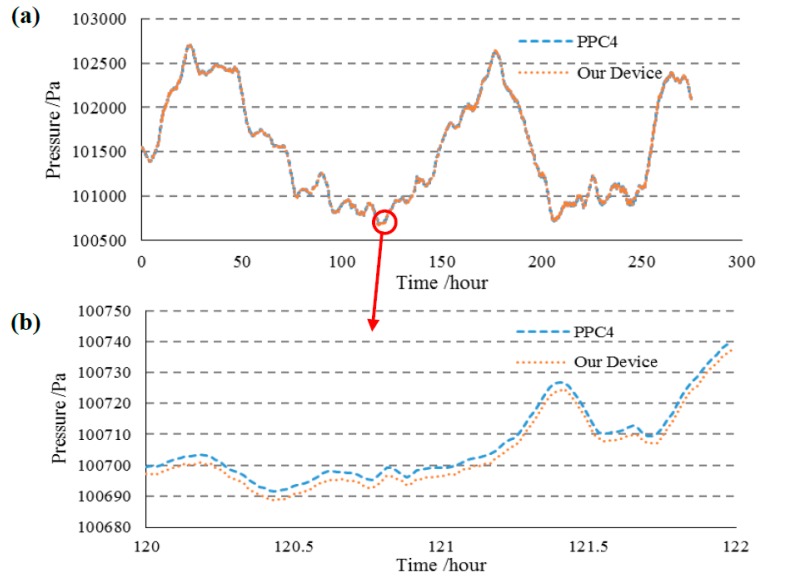
The actual atmospheric testing for a time duration of 270 h. (**a**) The device proposed in this study demonstrated comparable results with the pressure controller/calibrator during over 270 h of testing in the actual atmosphere; (**b**) The detailed experiments results where deviations lower than 8 Pa were observed.

Furthermore, the sensors were tested under actual atmospheric conditions for long-term stability quantification (a total of 270 h). Figure 7a shows that the device developed in this study had a very similar result (after temperature compensation) as the pressure controller/calibrator during several days of testing in the actual atmosphere. Figure 7b shows the detailed results in the time duration from 120 h to 122 h, where no deviation larger than 8 Pa between the pressure controller/calibrator and the pressure microsensor demonstrated in this study was observed.

## 6. Conclusions

This paper presented a resonant pressure microsensor capable of self-temperature compensation. Resonant pressure microsensors with two doubly-clamped “H” type resonant beams were fabricated and characterized. The algorithm enabling the calculation of temperature and pressure based on two resonant frequencies was proposed and optimized. After compensation, the errors were less than 0.01% of the full pressure scale (temperature range of −40 °C to 70 °C and pressure range of 50 kPa to 110 kPa), and thus the resonant pressure microsensor can work in the self-temperature compensation mode.
